# Validation of a frailty index in older cancer patients with solid tumours

**DOI:** 10.1186/s12885-018-4807-6

**Published:** 2018-09-14

**Authors:** A. L. McCarthy, N. M. Peel, K. M. Gillespie, R. Berry, E. Walpole, P. Yates, R. E. Hubbard

**Affiliations:** 1 School of Nursing, University of Auckland, Private Bag 92019, Auckland Mail Centre, Auckland, 1142 New Zealand; 2 School of Nursing, Queensland University of Technology, Victoria Park Rd, Kelvin Grove, QLD 4059 Australia; 3 Cancer Services, Princess Alexandra Hospital, 199 Ipswich Rd, Woolloongabba, QLD 4102 Australia; 4 Centre for Research in Geriatric Medicine, University of Queensland, Level 2, Building 33, Princess Alexandra Hospital, 199 Ipswich Rd, Woolloongabba, QLD 4102 Australia

**Keywords:** Geriatric oncology, Frailty, Comprehensive geriatric assessment, Chemotherapy

## Abstract

**Background:**

Frailty is an indicator of physiological reserve in older people. In non-cancer settings, frailty indices are reliable predictors of adverse health outcomes. The aims of this study were to 1) derive and validate a frailty index (FI) from comprehensive geriatric assessment (CGA) data obtained in the solid tumour chemotherapy setting, and 2) to explore whether the FI-CGA could predict chemotherapy decisions and survival in older cancer patients with solid tumours.

**Methods:**

Prospective cohort study of a consecutive series sample of 175 cancer patients aged 65 and older with solid tumours. A frailty index was calculated using an accumulated deficits model, coding items from the comprehensive geriatric assessment tool administered prior to chemotherapy decision-making. The domains of physical and cognitive functioning, nutrition, mood, basic and instrumental activities of daily living, and comorbidities were incorporated as deficits into the model.

**Results:**

The FI-CGA had a right-skewed distribution, with median (interquartile range) of 0.27 (0.21–0.39). The 99% limit to deficit accumulation was below the theoretical maximum of 1.0, at 0.75. The FI-CGA was significantly related (*p* < 0.001) to vulnerability as assessed by the Vulnerable Elders Survey-13 and to medical oncologists’ assessments of fitness or vulnerability to treatment. Baseline frailty as determined by the FI-CGA was also associated with treatment decisions (Treatment Terminated, Treatment Completed, No Planned Treatment) (*p* < 0.001), with the No Planned Treatment group significantly frailer than the other two groups.

**Conclusion:**

The FI-CGA is a potentially useful adjunct to cancer clinical decision-making that could predict chemotherapy outcomes in older patients with solid tumours.

## Background

Research interest in recent years has focused on methods to assess older cancer patients’ fitness for chemotherapy and to predict their potential responses to it [1–3]. Accurate prediction of chemotherapy outcomes could obviate current concerns about over- and under-treatment in this cohort [4–6]. An approach that has received consistent attention is comprehensive geriatric assessment (CGA). In this paper we report the results of a prospective cohort study. The aim of the study was to derive and validate a frailty index (FI) from comprehensive geriatric assessment (CGA) data obtained in the solid tumour chemotherapy setting.

‘Frailty’ is the accumulation of multiple physical and psychosocial deficits in the older person [7]. Frail older people have diminished capacity to compensate for stressors compared to people of the same chronological age; implying a state of elevated risk in the context of treatment decision-making [8]. The frailty index (FI), which is a continuous measure ranging from 0 to a theoretical maximum of 1.0, is calculated by dividing the number of deficits identified in the CGA by the total number of variables measured. The FI therefore signifies the extent of deficit accumulation: the more deficits that are accumulated, the more likely that the individual is frail. For example, if a CGA measures 50 separate variables, an older person who records 10 variables as deficits will have a FI-CGA of 10/50 (or 0.2) [9]. The FI has a theoretical maximum of 1.0 [10].

The FI-CGA has many advantages. First, unlike many measures of frailty (such as the Fried Phenotype) [11] the FI-CGA does not privilege physiological variables. Like the CGA from which it is derived, it is multidimensional, articulating the psychosocial aspects of risk as much as the physical aspects [12]. This is critical in the chemotherapy context where the contribution to treatment outcomes of variables such as cognitive function, mood and level of social support could have as much weight as comorbidities. A further advantage of the FI-CGA is its flexibility: assuming the instruments used to measure variables are validated and reliable, the index can be constructed from different numbers and types of variables that are relevant to specific contexts. Research suggests that, provided a minimum number of deficits are chosen (about 30), variables can be selected at random and still yield comparable results on risks of adverse outcomes [13]. Third, irrespective of the composition of the CGA, its variables can contribute to a summary outcome measure, usually taken as continuous, that could flag the degree of risk associated with standard chemotherapy treatment and thereby enable chemotherapy to be tailored to the older person’s identified deficits. The advantage of this is precision: the FI-CGA is far more precise than the dichotomous or interval-level risk stratifications that are common in the geriatric oncology field.

An FI was recently generated from CGA data (*n* = 1418) in the acute hospital setting, demonstrating that every 0.1 increase in the FI-CGA was associated with a doubling of mortality (OR: 2.05 [95% CI 1.78–2.48]) [14]. The construct validity of the FI-CGA was further confirmed in the chronic kidney care context (*n* = 110) [8], where the FI-CGA increased according to the severity of kidney disease (*p* = 0.04). In the study reported in this paper, we used CGA data to generate an FI relevant to the acute chemotherapy setting for the first time.

## Methods

### Aim

The overall aim of this prospective cohort study was to determine whether a clinically feasible and useful FI-CGA could be developed to predict chemotherapy decisions and survival in older patients with solid tumours.

The objectives were, in patients aged 65 years and over, to:

1. Develop an FI derived from a standardised comprehensive geriatric assessment process.
2. Compare FI-CGA assessments with:
  - Assessments of ‘fit’ and ‘vulnerable’ derived from the Vulnerable Elders Survey-13 [15].
  - Assessments of ‘fit’, ‘vulnerable’ and ‘frail’ derived from blinded physician judgements.
  - Documented treatment decisions (dose alterations during treatment, treatment completion), and survival over a period of 3.5 years of follow-up from date of CGA assessment.
3. Determine the clinical feasibility and utility of the CGA-derived FI in the oncology context.

Ethical approval was obtained from the Human Research Ethics Committee (HREC) - Metro South Health Service District (HREC/09/QPAH/269) prior to undertaking the study, and approval from the relevant statutory authority to obtain death registry data (where this was not recorded in the medical record). The study was undertaken according to the Strengthening the Reporting of Observational studies in Epidemiology (STROBE) guidelines [16].

### Setting and participants

Princess Alexandra Hospital is a publicly-funded teaching hospital in Brisbane, Australia. It services a large geographic area of urban, rural and remote Queensland, providing chemotherapy to approximately 2000 individual cancer patients each year. Consecutively-recruited eligible patients were aged 65 years or over at the time of diagnosis; were diagnosed with a new primary or recurrence of a solid tumour; were referred for potential chemotherapy; and were able to understand conversational English. Treatments administered in addition to chemotherapy (e.g. radiotherapy and haematopoietic stem cell or bone marrow transplants) complicate treatment decision-making. Hence patients were not eligible if they had a blood cancer, were scheduled to undergo chemo-radiation, or were, in the opinion of the treating oncologist, unable to provide informed consent. Participants were recruited before treatment decisions had been made, therefore eligible participants enrolled in the study may have been prescribed a range of chemotherapy regimens (including curative and palliative regimens, first- and second-line treatments), or no treatment.

### Procedure

Participants were recruited over 30 months from mid-2013 to December 2015, with longitudinal data collection ceasing by December 2016. Patients were identified by their treating oncologist and referred to the project officer, who screened for eligibility and obtained informed consent. The CGA was administered by a nurse extensively trained in geriatric assessment. The ideal in geriatric oncology is a tool that can predict chemotherapy outcomes at baseline and guide physicians and patients in their subsequent treatment decisions. Hence the CGA was administered once only, before the patient commenced treatment. Other baseline and prospective medical data including scheduled chemotherapy, chemotherapy modifications per cycle, treatment outcomes (scheduled therapy completed/not completed) and survival for up to 3.5 years of follow-up were drawn from the electronic medical record.

To obtain the blinded oncologists’ assessments of fitness for chemotherapy, the electronic chemotherapy prescribing system was configured with a pop-up questionnaire asking the oncologist to notate whether they believed, on the basis of their clinical examination, the patient was ‘fit’ for, ‘vulnerable’ to, or too ‘frail’ for standard adult chemotherapy. The oncologists were briefed on the precise meaning of these operational definitions, which were also built in to the pop-up questionnaire. Table 1 outlines the operational definitions used in the study.

**Table 1:** Operational definitions

Variable	Definition
Fit	Should tolerate standard adult cancer therapy in addition to the anti-emetic, growth factor, superhydration and other supportive therapies usually scheduled with standard adult cancer therapy, with no modification or abandonment of the prescribed regimen.
Vulnerable	Not likely to tolerate standard adult cancer therapy without requiring subsequent modification or abandonment of the prescribed regimen, but should tolerate an individually tailored anti-cancer treatment, plus supportive therapies. This might include treatment on an inpatient basis, and/or molecular-targeted therapy and/or reduction of cytotoxic drug in terms of dose, cycle or frequency at the oncologist’s discretion.
Frail	All functional reserves invested in basic survival, patient may not have any additional resources to cope with the stressors induced by cancer treatments. Hence supportive, palliative, molecularly-targeted and hormone modification therapies are not precluded; however high-toxicity therapies should be excluded.
Standard adult dose (SAD)	The facility where the study was conducted has well-defined chemotherapy guidelines developed by consultant oncologists with reference to the latest meta-analyses, systematic reviews, randomised controlled trials, and international and national guidelines. The SAD for each drug in each protocol is determined with reference to these resources using predefined weight-based, body surface area-based, or absolute or renal function-based dose rates. Each hospital cancer protocol is recorded in, and administered according to, the electronic chemotherapy prescribing system to ensure facility standardisation and consistent protocol delivery.
Dose alteration	Any anticancer drug in any regimen that was altered by 10% or more during the course of treatment.
Treatment completion	Is dichotomised to represent patients who: 1. Completed all cycles of treatment prescribed at baseline, or 2. Were not prescribed treatment due to patient decision or patient health, or withdrew prematurely from treatment due to toxicity, death, or patient decision.

### Measures

The CGA instruments used in this study are detailed in Table 2. A Frailty Index (FI) was calculated from CGA data based on the accumulated deficits model [13] using a well-defined methodology [8]. Variables can be considered as deficits and contribute to the FI if they are associated with health status, cover a range of systems, and their prevalence generally increases with age but does not saturate (i.e., have a high prevalence so as to be almost ubiquitous) [14]. In summary, data collected as part of the CGA assessment were coded as deficits across domains of physical and cognitive functioning, nutrition, mood, basic and instrumental activities of daily living, and comorbidities (total number of deficits = 42) to construct an FI based on the CGA (FI-CGA).

**Table 2:** Creation of Frailty Index from assessment tools

Assessment tools	Rigour in older cancer patients	Variables used to code deficits for FI	Deficit Total
Cumulative Index Scale-Geriatrics (CIRS-G)	Confirmed criterion validity [27], good concurrent construct and validity [27], intrarater reliability *r* = 0.83 and interrater reliability *r* = 0.81[27, 28]	• Comorbidities coded as deficit (=1) for each comorbidity up to a maximum of 12	12
Malnutrition Screening Tool (MST)	Sensitivity 100% [29]; specificity 81–92% [29]; PPV 0.4–0.8[29]; NPV = 1.0[29]; IRR good [29]	• BMI Weight (kg)/Height (m^2^) outside normal range (< 22 or > 27) = 1 • Weight loss ≥6 kg = 1 • Decreased appetite = 1	3
Standardised Mini-mental State (SMMSE)	Reliability α = 0.65–0.732[30]; sensitivity 91% [31]; specificity 91% [31]	• Cognitive impairment - Mild (MMSE 20–25) = 0.5 - Moderate/severe (MMSE< 20) = 1	1
Geriatric Depression Scale (GDS)	Sensitivity 96% [31] and specificity 88% [31]	• GDS score ≥ 5 = 1	1
Modified Barthel Index (MBI)	Criterion and construct validity established [32]; test-retest reliability *r* = 0.7[32]; inter-rater reliability *r* = 0.99[32]	• ADL (personal hygiene, bathing, feeding, toileting, stairs, dressing, ambulation, transfers) - Minimal help required = 0.5 - Moderate help required /Unable to do = 1	8
		• Continence (bowel and bladder) - Minimal help required = 0.5 - Moderate help required /Unable to do = 1	2
Lawton IADL Scale (IADL)	Inter-rater reliability *r* = 0.85[33]; reliability α = 0.86[33]	• IADL (phone use, shopping, food preparation, house- keeping, laundry, transport, medications, finances) - Minimal help required = 0.5 - Moderate help required /Unable to do = 1	8
Vulnerable Elders Survey-13	Validity established [15]; reliability α = 0.90[15]; sensitivity 90% and specificity 70% [15]	• Self-rated health fair/poor = 1	1
		• Physical performance measures (stooping, lifting, reaching, writing, walking, housework) - A little/some difficulty - A lot of difficulty/Unable to do	6

Participants were classified as Vulnerable/Not Vulnerable using the Vulnerable Elders Survey-13 (VES-13), a validated measure to identify older people at risk of functional decline and death [15]. The treating oncologist also assessed participants as Fit, Vulnerable or Frail based on the operational definitions outlined in Table 1. Treatment decisions comprised dose alterations of > 10% during treatment and treatment completion, with the remaining treatment outcome comprising median survival. These terms are operationally defined in Table 1.

Demographics (age, gender and socioeconomic status, living arrangements and social support such as home help) and chart data including body mass index, concomitant medications and tumour type and stage were collected. All patients were followed-up for up to 3.5 years (depending on the date of their enrolment in the study) through chart data and consultation with the State death registry after baseline assessment to determine survival.

### Data analysis

Data were analysed using the Statistical Package for the Social Sciences version 23.0 [17]. Frequency distributions were used to describe the data, with proportions calculated as percent of available data. For concurrent validity, the relationship of the FI-CGA to other measures of vulnerability (VES-13 or Doctor Assessment) was tested using parametric or non-parametric tests, depending on distribution of the data, and effect sizes calculated (r = Z/sqrt[N]) and interpreted [18]. To investigate the predictive ability of the baseline FI on treatment decisions, regression (logistic or ordinal) was performed, adjusting for age and gender, to calculate an odds ratio (OR) with 95% confidence interval (CI). The FI was categorised into groups at 0.1 intervals for ease of interpretation of the OR as the likelihood of the outcome for each 0.1 increase in the FI [19]. Kaplan Meier survival analysis assessed cumulative survival by frailty group, dichotomised as ‘fit’ or ‘frail’ and based on an established FI-CGA cut-point of ≤0.25 [13].

## Results

One hundred and seventy five consecutively recruited patients consented to the CGA process. Participants were predominantly male (*n* = 108/61.7%), with females making up roughly one third of the cohort (*n* = 67/38.3%). The mean age was 72.0 years (SD = 5.2, Range = 65–86). The majority of participants were married or living in a de facto relationship (*n* = 122/69.7%), with the remainder not partnered. Almost all participants were pensioners (*n* = 157/89.7%), though a small number were employed (*n* = 9/5.1%) or self-funded retirees (*n* = 8/4.6%). Lung tumours (*n* = 51, 29.1%) and colorectal tumours (*n* = 42, 24.0%) were the most frequent cancer diagnoses recorded. The mean time to death was 9.15 months (SD = 7.712), range 0 to 33 months.

The FI-CGA could be calculated on all patients by adjusting the denominator to account for missing data. In this study 154 (88%) had complete data (denominator = 42), 19 (11%) had 1 missing deficit component (denominator = 41) and 2 patients had denominators of 40 and 39, to account for missing data. The index had a right-skewed distribution, with mean (SD) of 0.31 (0.14) and median (IQR) of 0.27 (0.21–0.39). Only 1% of the sample had an FI-CGA more than 0.75. Similar limits to deficit accumulation have been reported in community-dwelling and inpatient populations, suggesting that there is a demonstrable ceiling to the number of health problems that people can tolerate [20]. While the mean FI increased by age group (65–69: 0.29; 70–74: 0.31; 75–79: 0.32; ≥80: 0.33) these differences were not significant, nor were there significant differences based on sex. Characteristics of the patients are shown in Table 3.

**Table 3:** Characteristics of the study sample

Characteristic	Total *N* = 175
Age mean (SD)	72.0 (5.2)
Age groups	
– 65–69	72 (41.1)
– 70–74	46 (26.3)
– 75–79	41 (23.4)
– ≥80	16 (9.1)
Sex	
– Males	108 (61.7)
– Females	67 (38.3)
Comorbidities median (IQR)	7 (5–7)
BMI median (IQR)	26.2 (23.1–30.6)
Malnutrition (MST) range 0–5	
– Low risk (0–1)	82 (46.9)
– Medium risk (2–3)	69 (39.4)
– High risk (4–5)	24 (13.7)
Cognition (SMMSE) range 0–30	
– Normal (26–30)	156 (89.7)
– Mild impairment (20–25)	15 (8.6)
– Moderate impairment (14–19)	2 (1.1)
– Severe impairment (< 14)	1 (0.6)
Depression (GDS) range 0–15	
– Little/no risk (< 5)	122 (70.9)
– Probable risk (≥ 5)	50 (29.1)
Modified Barthel Index median (IQR)	98 (93–100)
Frailty Index median (IQR)	27 (0.21–0.38)
Fit (FI ≤ 0.25)	81 (46.3)
Frail (FI > 0.25)	94 (53.7)

Figures represent number (%) unless otherwise specified

As shown in Table 4, descriptive statistics showed that the Vulnerable group as measured by the VES-13 had higher median (IQR) FI (0.42 [0.36–0.52]) than the Not Vulnerable group (0.24 [0.19–0.29], Mann-Whitney U = 679.5, Z = − 8.86, *p* < 0.001, *r* = − 0.67). For the clinician assessment, a Kruskal-Wallis test was found to be statistically significant (χ^2^ (2) = 27.4, p < 0.001). Post hoc-tests (with Bonferroni correction) showed that the median (IQR) effect size for FI between Fit (0.24 [0.19–0.32]) and Vulnerable (0.30 [0.22–0.43]) was small (*r* = 0.29) although significant (*p* = 0.001) and between Fit ([0.24 (0.19–0.32]) and (Frail 0.38 [0.29–0.61]) was medium (*r* = 0.43) and also significant *p* < 0.001.

**Table 4:** Association with frailty index

Assessments	*N* = 175	FI median (IQR)	Effect Size	*p* value
VES Assessment				
Not vulnerable	112 (64.0%)	0.24 (0.19–0.29)	*r =* 0.67	< 0.001
Vulnerable	63 (36.0%)	0.42 (0.36–0.52)		
Dr Assessment				
Fit	90 (53.3%)	0.24 (0.19–0.32)	Reference	
Vulnerable	52 (30.8%)	0.30 (0.22–0.43)	*r* = 0.29	0.001
Frail	27 (16.0%)	0.38 (0.29–0.61)	*r* = 0.43	< 0.001
Treatment Decisions	*N* = 175	FI median (IQR)	OR (95% CI)	
Treatment plan				
Completed	46 (26.9%)	0.24 (0.15–0.35)	1.65 (1.32–2.05)	< 0.001
Terminated	94 (55.0%)	0.27 (0.21–0.36)		
Not planned	31 (18.1%)	0.40 (0.30–0.56)		
Drug alteration				
< 10%	87 (61.7%)	0.27 (0.21–0.35)	0.98 (0.71–1.28)	0.89
≥ 10%	54 (38.3%)	0.26 (0.20–0.38)		

In ordinal regression, baseline frailty as determined by the FI-CGA was also associated with treatment plan (OR 1.65; 95% CI 1.32–2.05). Those who completed the treatment plan (highest level) were the least frail (FI median (IRQ): 0.24 [0.15–0.35]), while those whose treatment plan was terminated (middle level) (FI median (IRQ): 0.27 [0.21–0.36]), or not planned (lowest level) (FI median (IRQ): 0.40 (0.30–0.56)) were progressively frailer. There was no significant relationship between change in standard adult dose during treatment and FI-CGA (Table 4).

**Fig. 1:**
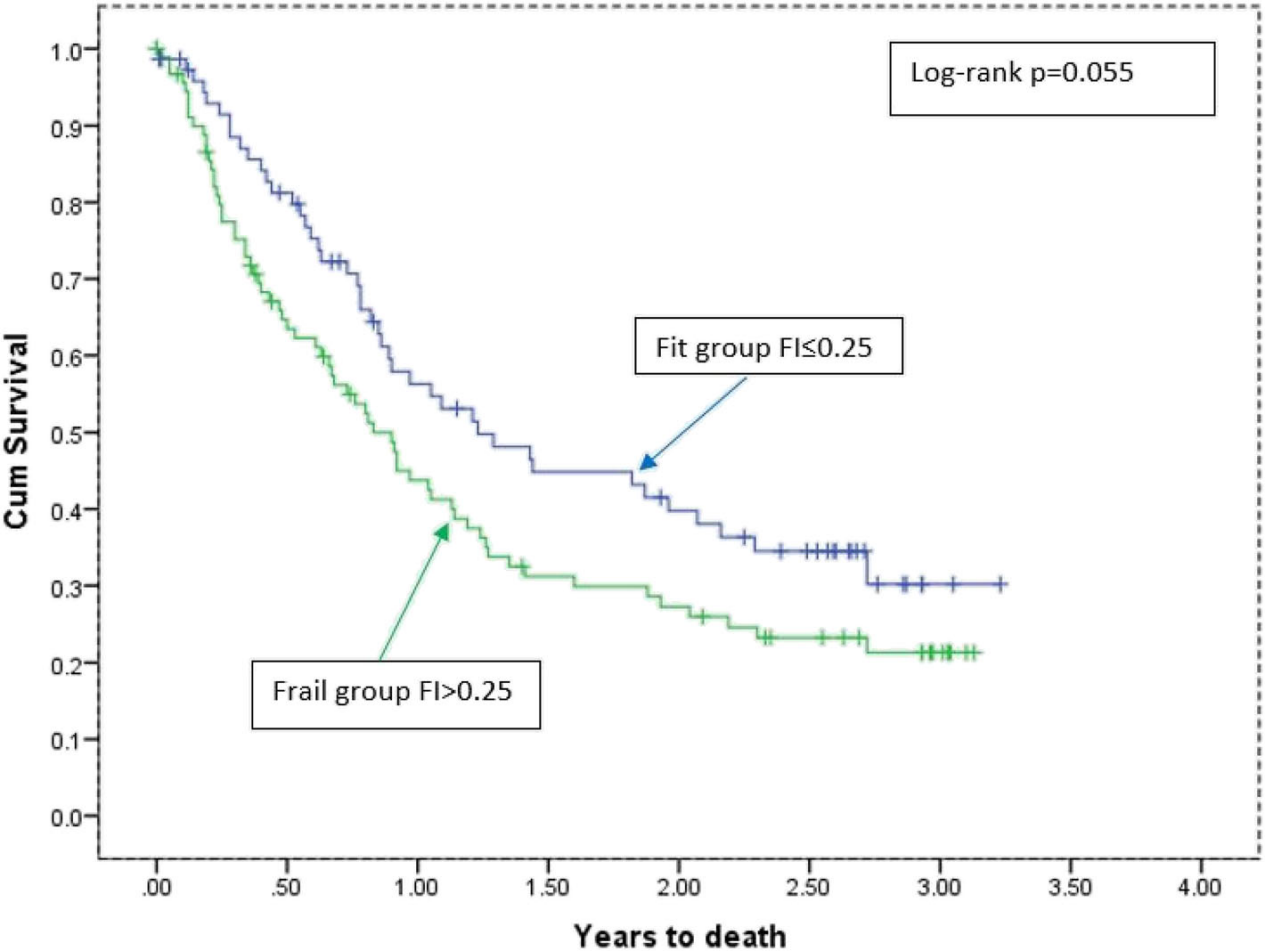
Survival analysis

Of the 174 patients with follow-up data, 108 died during the follow-up period, 77 of whom died in the first year post-assessment. Figure 1 indicates a trend for better cumulative survival in the ‘fit’ compared with the ‘frail’ group using the FI-CGA cut-point of ≤0.25 to categorise the ‘fit’ group [13] (Log Rank (Mantel-Cox) = 3.67; *p* = 0.055).

## Discussion

The results demonstrate that the FI-CGA is a clinically feasible tool to predict chemotherapy outcomes in older patients with solid tumours. The No Plan group was significantly frailer than the Treatment Terminated or Treatment Completed groups (i.e., those patients at baseline who were, in medical opinion, able to tolerate chemotherapy). There was also a trend for patients classified as frail by the FI-CGA to be more likely to die in the study follow-up period. The FI-CGA also demonstrated good construct validity against fitness and vulnerability as measured by the VES-13, which has previously been shown to be highly predictive of impaired functional status in older patients with cancer [21]. There was also a significant association between subjective assessments of frailty by the treating oncologists compared with objective measurement of frailty using the FI-CGA. We acknowledge that this result in this study is an outcome of experienced oncologists working in a highly-specialised tertiary facility. Subjective assessment or ‘eyeballing’ patients to estimate frailty status, however, is generally fraught with difficulties and tends to be inconsistent [22].

Several good studies have tested whether elements of the CGA can rapidly predict selected chemotherapy outcomes such as toxicity [1, 3, 23]. To the best of our knowledge, this is the first study to derive an FI from the CGA to predict a range of significant clinical outcomes in older chemotherapy patients. A great deal of research in this cohort has also focused on rapid screening of frailty in order to select patients who need much more detailed assessment by way of CGA [24]. However, these simple frailty screening measures are not considered to have the discriminative ability to identify patients who might need further assessment before chemotherapy decisions are made [24, 25].

The FI-CGA demonstrated adequate discriminative ability in the study context, in that it identified patients for whom cancer treatment was also not deemed medically-appropriate. Our approach of deriving an FI from the CGA might therefore be useful in other oncology programs where treatment planning decisions are needed. It should be emphasised that the FI-CGA should not be used to discriminate against patients being assigned cancer treatment. Rather, it should be used to identify patients who might lack the reserves to cope with standard adult chemotherapy and who might benefit from individually-tailored supportive therapies, or geriatrician and allied health input before treatment decisions are made. In our clinical experience, these approximate to the 36% of participants who crudely screened as ‘vulnerable’ by way of the VES-13; the 31% deemed ‘vulnerable’ by physicians before treatment commenced; and the 33% scoring between > 0.25 to 0.4 on the FI continuum. This group is perhaps the most at-risk in terms of under- or over-treatment, and most in need of careful treatment decisions. Using the FI-CGA to flag these older patients at-risk, and exploring evidence-based treatment algorithms and referral pathways for this group, features in our research agenda.

Importantly, it is possible to derive the FI rapidly once a CGA is undertaken. For instance, it has recently been demonstrated that an FI can be derived from routinely-collected patient electronic records [26]. Accordingly, using an electronically derived FI-CGA would be particularly advantageous in the oncology setting if CGA is already standard practice.

The strength of this study is that CGAs were available for all study participants and hard clinical outcomes relating to the toleration of chemotherapy, not just mortality, were collected. Like all studies, it had limitations. Collection of data through chart review might have underestimated some outcomes. For example, cancer staging, which could have affected the outcomes, could not be accounted for in the analysis, because these data were often not documented in the medical record. However, ready access to the linked State death registry ensured that mortality outcomes were accurate when death was not documented in the patient record. While a pragmatic decision was made to include all-comers in this relatively small sample from one clinic to reflect the reality of our clinical practice, a more rigorous design would restrict the sample to one tumour type, chemotherapy-naïve patients and preferably, to one chemotherapy protocol. Due to relatively small sample size it was not possible to adjust for possible confounders, which could have biased study results and limited the generalisability of the study. Nonetheless, because of its ability to be derived from any CGA, the FI-CGA can potentially be applied across multiple oncology settings such as surgery, radiotherapy and haematology. In turn, this will facilitate comparative research.

## Conclusion

Despite their over-representation in cancer populations, the older person’s cancer treatment needs are not well-understood. They are often at risk of over- and under-treatment. Precise determination of risk in older people being considered for chemotherapy is critical to the development of individualised care pathways and management algorithms. Frailty is an excellent indicator of the older patient’s physiological reserves. The results of this exploratory work indicate that a frailty index derived from CGA inputs can potentially contribute to accurate assessment of treatment outcomes in the chemotherapy setting.

## Abbreviations

CGA: Comprehensive geriatric assessment
FI: Frailty index
HREC: Human Research Ethics Committee
STROBE: Strengthening the Reporting of Observational Studies in Epidemiology
VES-13: Vulnerable Elders Survey-13
